# Diagnostic value of urine cyclic RNA-0071196 for bladder urothelial carcinoma

**DOI:** 10.1186/s12894-024-01466-z

**Published:** 2024-04-16

**Authors:** Yang Yang, Jun Li, Weixiang Yao, Ge Zou, Xuying Ye, Qishan Mo

**Affiliations:** Department of Urology, Panyu District Central Hospital, No.8 Fuyu East Road, Guangzhou, 510000 China

**Keywords:** Bladder urothelial carcinoma, Circular RNA-0071196, Analysis of clinical case characteristics

## Abstract

**Objective:**

To investigate the diagnostic value of urine cyclic RNA-0071196 (circRNA-0071196) in the patients with bladder urothelial carcinoma (BUC).

**Method:**

The expression of circRNA-0071196 was detected in the urine samples using qRT-PCR from 40 BUC patients and 30 non-UBC patients at our department from December 2018 to September 2021. The expression difference of circRNA-0071196 was compared between the two groups, and the relationship between the expression of circRNA-0071196 in the urine of UBC patients and the clinical pathological characteristics was analyzed.

**Results:**

(1) The expression of circRNA-0071196 in the urine of BUC group was significantly higher than that in the non-BUC group (*P* < 0.05). (2) The expression of circRNA-0071196 in the urine of BUC group was not related to age, sex, or lymph node metastasis (*P* > 0.05). (3) The expression of circRNA-0071196 in the urine of BUC group was related to tumor T stage, tumor grade and muscle invasion. (4) The urine circRNA-0071196 expression effectively distinguished BUC patients from non-BUC patients.

**Conclusion:**

The elevated expression of urine circRNA-0071196 in BUC patients indicates that circRNA-0071196 has promising potential as a non-invasive urinary biomarker for detecting BUC.

## Background

Bladder cancer has emerged as a prevalent malignant tumor in the urinary system worldwide. Histologically, bladder urothelial carcinoma (BUC) accounts for approximately 90% of all bladder cancer cases. In 2020, bladder cancer ranked tenth among all malignancies in terms of incidence, and it stood second in both morbidity and mortality among male urinary malignancies [1]. The global number of bladder cancer patients has exceeded 400,000, with a higher proportion of male patients and a greater likelihood of occurrence among individuals aged over 60. Notably, individuals under the age of 40 are more susceptible to bladder cancer due to increased exposure to toxic substances like nicotine, which is also a major cause of this disease [2, 3]. Tissue biopsy via cystoscopy remains the gold standard for diagnosing bladder cancer. Although gold immunochromatographic assays for nuclear matrix protein 22 and bladder tumor antigen have been approved by the US Food and Drug Administration as noninvasive clinical methods, their limited sensitivity and proneness to false positives in the presence of hematuria pose significant challenges [4–6]. Therefore, there is an urgent need for a diagnostic method that balances sensitivity and specificity in bladder cancer.

Circular RNA, consisting of closed RNA molecules formed by connecting the 3’ and 5’ ends, exhibits diverse sequence compositions and can be formed by exons or introns. Numerous studies indicate their crucial roles in various biological processes [7]. While the specific functions of circRNA remain incompletely understood, current research suggests that they serve as “molecular sponges” for microRNAs (miRNAs), interacting through competitive binding, and indirectly influencing the expression of downstream target genes, thus contributing to complex regulatory mechanisms [8, 9]. It has been observed that the expression level of circular RNA in urine varies across multiple diseases, offering potential opportunities for circular RNA as a novel diagnostic and therapeutic approach. Unlike other sampling methods, urine collection is a noninvasive, painless, and highly convenient technique that enhances the accuracy and effectiveness of early disease diagnosis and treatment [10]. Urinary cytology test can be used as the gold standard for the diagnosis of bladder cancer, but this method has insufficient cell collection or improper treatment, which may lead to too low a number of cancer cells in the sample, making them difficult to detect and prone to false negatives [11, 12]. Additionally, Urine cytology tests are judged by experienced pathologists, while circRNA in urine can be stably present in urine, and can be detected by PCR even in minute quantities, with the test results being less susceptible to human factors. We believe that urine cytology and circular RNA testing have their own characteristics and should be used in combination. Meanwhile, the clinical diagnostic value of circular RNA in the urine of bladder cancer patients requires further exploration.

Therefore, this study investigates the overexpression of circRNA-0071196 in BUC samples based on previous studies, and further explored relationship between the expression of urine circRNA-0071196 and clinicopathological characteristics in BUC patients, aiming to explore the potential of urine circRNA-0071196 as a biomarker of BUC.

## Materials and methods

### Experimental grouping and specimen processing

Urine samples from 40 BUC patients and 30 non-UBC patients were collected from Guangzhou City Panyu Central Hospital. People in the non-bladder cancer group were healthy volunteers without a family history of bladder cancer or exposure to risk factors for bladder cancer. All samples were verified histopathologically. All urine samples were stored in a refrigerator at-80℃ until further use. This study has been approved by the Ethics Committee of Guangzhou City Panyu Central Hospital.

### Total RNA extraction and qRT-PCR

RNA was extracted from urine samples by TRizol method. The concentration and purity of extracted RNA were detected by Nanodrop 2000 micro-ultraviolet spectrophotometer (NanoDrop, Wilmington, DE, USA). Total RNA was digested with RNase R (Epicentre, USA) to remove linear RNA and enrich circRNA concentration and purity before reverse transcription. Reverse transcription was performed using SuperScript™III Reverse Transcriptase Kit (Invitrogen/Thermo Fisher Scientific) according to the instructions for use. Real-time quantitative PCR was performed using Step One Plus PCR instrument (Applied Biosystems, Bedford, MA, USA), reaction volume was 20 µL, reaction conditions: 95°C for 10 minutes, then 40 cycles of 95°C for 10 seconds, 55°C for 30 seconds annealing. PCR was performed using the Light Cycler 96 and data were analyzed using the ViiA 7 Real-time PCR System (Applied Biosystems, Bedford, MA, USA). Relative expression of circRNA-0071196 was calculated using 2 ^-ΔΔCt^ with GAPDH as the internal reference gene. The circRNA-0071196 primer sequence is: F:5’TGCTTATAGAAGGAACAAGCAG3’, R:5’TACTCATATTTGAAACCGGATCT3’; the GAPDH primer sequence is: F:5’TGACTTCAACAGCGACACCCA 3’, R: 5’CACCCTGTTGCTGTAGCCAAA 3’。.

### Voided urine cytology procedures

Voided urine cytology was carried out by a cytopathologist at our institution. Positive/suspicious cytology results were counted as positive and inconclusive/normal cytology results were classified as negative.

### Statistical analysis

Statistical analysis was performed using SPSS 17.0. Continuous variables were analyzed using Student*’s t-test*. The relative expression of circRNA-0071196 was expressed as mean ± standard deviation. Sensitivity (SN), specificity (SP), positive predictive value (PPV), negative predictive value (NPV) and accuracy were calculated according to standard statistical methods. Receiver operating characteristic curve (ROC) and area Under curve (AUC) were used to evaluate the diagnostic specificity and sensitivity of circRNA-0071196, The cut-off threshold of circRNA-0071196 was chosen based on the optimal combination of sensitivity and specificity, as derived from the analysis of the curve intersection.*P <* 0.05 was considered as significant difference.

## Results

### The expression of urine circRNA-0071196 between BUC and non-BUC groups

**Fig. 1:**
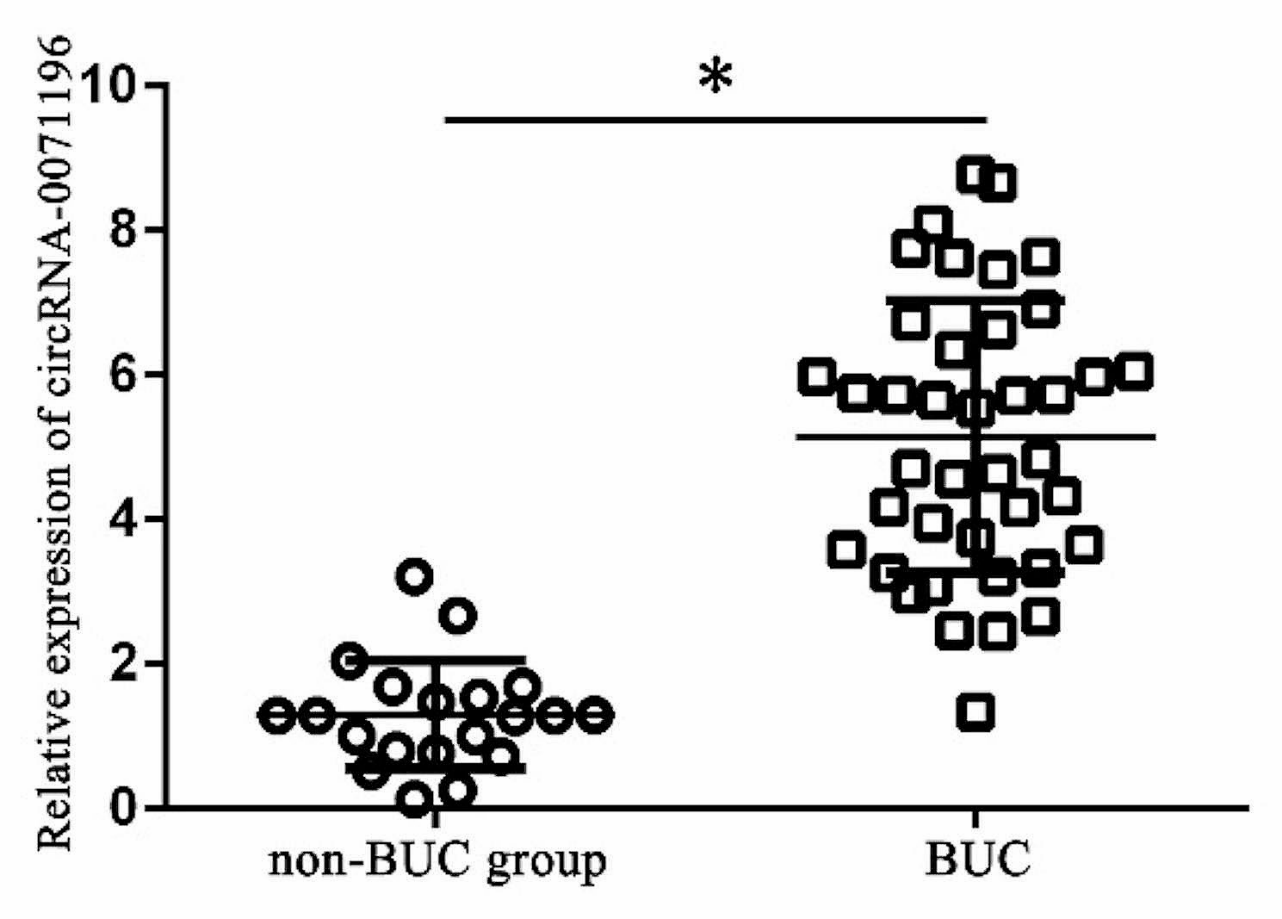
The relative expression of urine circRNA-0071196 in BUC and non-BUC groups. * *P* < 0.05

The expression of urine circRNA-0071196 in BUC group was up-regulated compared to non-BUC group (1.91 ± 1.148 vs. 5.18 ± 1.860, *P* < 0.05) (Fig. 1).

### The relationship between clinical features and urine circRNA-0071196 in BUC patients

**Fig. 2:**
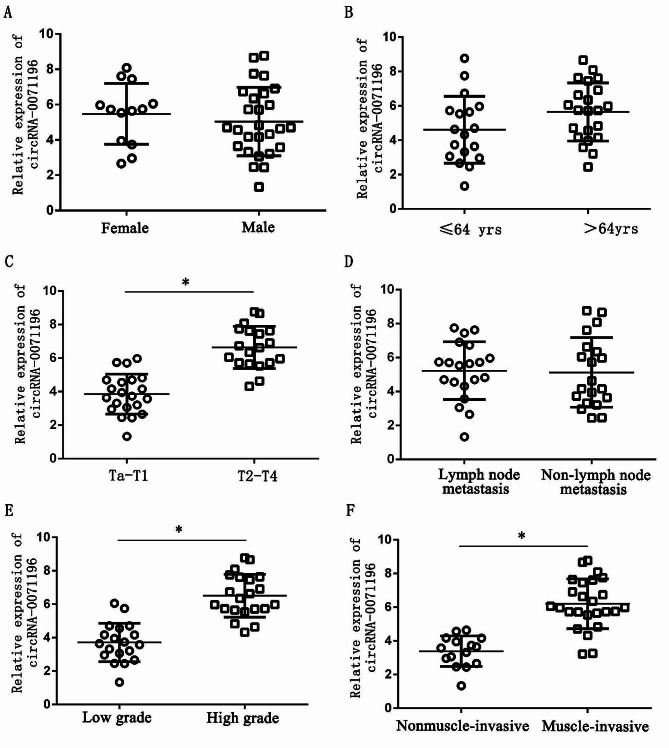
The relationship of Clinical features and urine circRNA-0071196 in BUC patients. Sex (**A**), age (**B**), T stage (**C**), lymph node metastasis (**D**), G stage (**E**), muscle invasion (**F**). * *P* < 0.05

The relative expression of urine circRNA-0071196 show no significant differences in terms of gender composition, age, and lymph node metastasis (*P* > 0.05) (Fig. 2A, B and D). However, there was a statistically significant correlation between the relative expression of urine circRNA-0071196 and T stage, G stage of bladder cancer, and muscle invasion (3.86 ± 1.195 vs. 6.64 ± 1.265, 3.71 ± 1.156 vs. 6.51 ± 1.258 and 3.37 ± 0.875 vs. 6.39 ± 1.253, *P* < 0.05) (Fig. 2C, E, F).

### Detection of urine circRNA-0071196 for diagnosing BUC

**Fig. 3:**
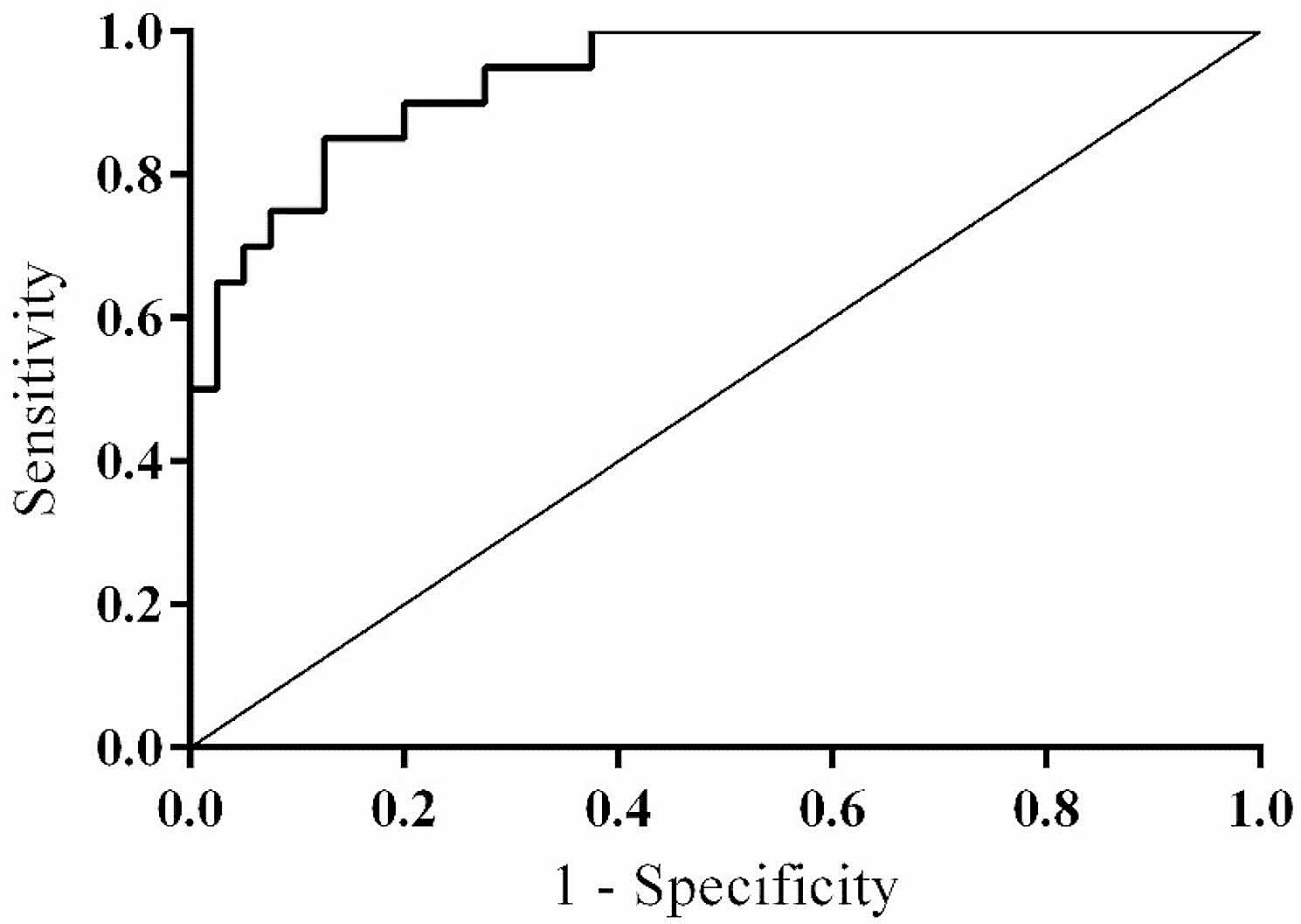
ROC curve of circRNA-0071196 for diagnosing BUC

The ROC curve was utilized to evaluate whether the expression of circRNA-0071196 could be used as a potential marker to assist in the diagnosis of patients with bladder cancer. The results demonstrated that urine circRNA-0071196 levels effectively differentiated patients in BUC group from those in the non-BUC group, with an AUC of 0.935 (95% CI: 0.876–0.994) (Fig. 3).

### Tumor diagnostic sensitivity between urine circRNA-0071196 and cytology

We next compared tumor diagnostic sensitivity between urine circRNA-0071196 and conventional cytology. The urine circRNA-0071196 test resulted in an SN of 87.5%, an SP of 85%, and an NPV of 77.3%, Cytological examination showed an SN of 70%, an SP of 95%, and an NPV of 61.3% (Table 1).

**Table 1:** Sensitivity, specificity, positive predictive value, negative predictive value and accuracy of the circRNA-0071196 test and urine cytology

	CircRNA-0071196 test	Urine cytology
Sensitivity (%)	87.5	70
Specificity (%)	85	95
Positive predictive value (%)	92.1	96.6
Negative predictive value (%)	77.3	61.3
Accuracy (%)	86.7	78.3

## Discussion

In recent years, there has been a notable increase in the incidence and mortality of bladder cancer in China. Despite undergoing treatments such as surgery, chemotherapy, and radiotherapy, patients with bladder cancer still face high recurrence rates and low 5-year survival rates [13, 14]. Given these challenges in treatment and prognosis, it is crucial to explore new biomarkers for bladder cancer while actively seeking new treatment methods. Identifying these biomarkers will enable physicians to accurately detect, identify, and analyze patients’ health conditions, facilitating the development of tailored and effective treatment strategies that can significantly improve cure rates and patient outcomes.

In our previous study, we employed gene chip technology to investigate the expression profiles of circRNAs in four cases of bladder cancer and matched paracancerous tissues. Through this analysis, we identified 127 differentially expressed circRNAs between bladder cancer and paracancerous tissues, including 89 up-regulated circRNAs and 38 down-regulated circRNAs. Our findings suggest that circRNA‑0071196 upregulates CIT levels in BCa by sponging off miRNA-19b-3p, subsequently influencing the proliferation, migration and colony formation capacity of the BCa cells [15]. There is evidence suggesting that the mechanism of action of circRNA involves acting as a “miRNA sponge.” In this process, circRNA competes with miRNA, leading to the inhibition of miRNA transcription and subsequent hindrance of mRNA transcription. Numerous studies have identified circRNAs capable of serving as miRNA sponges, highlighting their significance as important regulators of gene expression [16]. The investigation of urine circRNA represents an emerging research area. Urine circRNA can be stably present in urine and reflect in vivo pathophysiological changes. Increasing studies have demonstrated its potential as a biomarker for various diseases [17, 18]. Urine samples are easily collected non-invasively, making urine circRNA a promising tumor marker. Significant alterations in urine circRNA have been observed in colorectal cancer, prostate cancer, bladder cancer, pancreatic cancer, and other cancer types, suggesting their potential for early cancer diagnosis and prediction of clinical progression. While further validation is necessary, urine circRNA holds great promise as a significant research direction in the field of cancer screening, diagnosis, and treatment in the future [19–23].

Based on previous experimental results, circRNA-0071196 was selected as the focus of this study. The relationship between its relative expression in the urine of 40 BUC patients and the basic clinical characteristics was examined using qRT-PCR. The results (Fig. 2) indicated that there were no statistically significant differences in the relative expression of urine circRNA-0071196 based on gender composition, age, lymph node metastasis, etc. However, a significantly higher relative expression of urine circRNA-0071196 was observed in bladder cancer patients with T2-T4 stage compared to those with Ta + T1 stage. Additionally, the relative expression of urine circRNA-0007905 was significantly higher in high-grade compared to low-grade BUC patients in terms of the G stage. These findings suggest a potential association between circRNA-0071196 expression in urine and the differentiation and invasive growth of BUC.

In this study, the ROC curve was employed to evaluate whether the expression of urine circRNA-0071196 could serve as a potential auxiliary marker for the diagnosis of bladder urothelial cancer patients (Fig. 3). The urinary circRNA-0071196 assay was significantly more sensitive than cytology in detecting bladder cancer, but less specific than cytology (Table 1). The results demonstrated that the urine circRNA-0071196 expression effectively distinguished BUC patients from non-BUC patients, indicating its potential diagnostic value for BUC. In the future, the detection of urine circRNA-0071196 can be included in the diagnosis of BUC to better assess patient condition and monitor treatment effectiveness. In conclusion, this study successfully detected the expression of urine circRNA-0071196 in BUC patients, explored its correlation with clinical pathological features of BUC, and evaluated its potential as a diagnostic marker. These findings provide a novel approach to explore novel candidate tumor markers and therapeutic targets for BUC.

## Data Availability

The raw data supporting the conclusions of this article will be made available by the authors upon request.
